# Identification of 1-Methylnicotinamide as a specific biomarker for the progression of cirrhosis to hepatocellular carcinoma

**DOI:** 10.1007/s00432-024-05848-6

**Published:** 2024-06-18

**Authors:** Sijia Zhang, Ping Tuo, Yuanye Ji, Zuoan Huang, Zi Xiong, Hongshan Li, Chunyan Ruan

**Affiliations:** 1 Centre for Medical Research, Ningbo No. 2 Hospital, Ningbo, 315010 China; 2 Liver Disease Department of Integrative Medicine, Ningbo No. 2 Hospital, Ningbo, 315010 China

**Keywords:** Hepatocellular carcinoma, Biomarkers, 1-Methylnicotinamide, Untargeted metabolomics, Machine learning

## Abstract

**Purpose:**

Hepatocellular carcinoma (HCC) is a prevalent malignant tumor, often arising from hepatitis induced by the hepatitis B virus (HBV) in China. However, effective biomarkers for early diagnosis are lacking, leading to a 5-year overall survival rate of less than 20% among patients with advanced HCC. This study aims to identify serum biomarkers for early HCC diagnosis to enhance patient survival rates.

**Methods:**

We established an independent cohort comprising 27 healthy individuals, 13 patients with HBV-induced cirrhosis, 13 patients with hepatitis B-type HCC, and 8 patients who progressed from cirrhosis to hepatocellular carcinoma during follow-up. Serum metabolic abnormalities during the progression from cirrhosis to HCC were studied using untargeted metabolomics. Liquid chromatography-mass spectrometry-based metabolomics methods characterized the subjects’ serum metabolic profiles. Partial least squares discriminant analysis (PLS-DA) was employed to elucidate metabolic profile changes during the progression from cirrhosis to HCC. Differentially expressed metabolites (DEMs) between cirrhosis and HCC groups were identified using the LIMMA package in the R language. Two machine learning algorithms, Least Absolute Shrinkage and Selection Operator (LASSO), and Random Forest Classifier (RF), were used to identify key metabolic biomarkers involved in the progression from cirrhosis to HCC. Key metabolic biomarkers were further validated using targeted metabolomics in a new independent validation cohort comprising 25 healthy individuals and 25 patients with early-stage hepatocellular carcinoma.

**Results:**

A total of 155 serum metabolites were identified, of which 21/54 metabolites exhibited significant changes in HCC patients compared with cirrhosis patients and healthy individuals, respectively. PLS-DA clustering results demonstrated a significant change trend in the serum metabolic profile of patients with HBV-induced cirrhosis during the progression to HCC. Utilizing LASSO regression and RF algorithms, we confirmed 10 key metabolic biomarkers. Notably, 1-Methylnicotinamide (1-MNAM) exhibited a persistent and significant decrease in healthy individuals, cirrhosis, and HCC patients. Moreover, 1-MNAM levels in developing patients were significantly higher during the cirrhosis stage than in the HCC stage. Targeted metabolomic validation in an external cohort further confirmed the good diagnostic performance of 1-MNAM in early HCC detection.

**Conclusion:**

Our findings imply that 1-MNAM may be a specific biomarker for the progression of cirrhosis to HCC.

**Supplementary Information:**

The online version contains supplementary material available at 10.1007/s00432-024-05848-6.

## Introduction

Chronic hepatitis B is a deadly public health problem impacting over 71 million people. In China, Chronic hepatitis caused by hepatitis B virus (HBV) is considered to be the main cause of hepatocellular carcinoma (HCC) (Liaw 2006; Xia et al. 2022). Presently, HCC ranks as the third most common cause of cancer-related deaths, with mortality rates continuing to rise in developed countries (Cao et al. 2020; Ferlay et al. 2015).

The early clinical recessive symptoms of HCC make early diagnosis difficult for high-risk populations. Based on imaging alone, it remains difficult to distinguish premalignant lesions of HCC from cirrhotic nodules. In recent decades, substantial efforts have focused on identifying and validating optimal biomarkers as non-invasive tools for clinical decision-making in various contexts, including early diagnosis, follow-up, prognostic prediction, and treatment guidance (Siravegna et al. 2019). Alpha-fetoprotein (AFP) is closely associated with the occurrence of HCC and is a major serum marker for identifying and screening people at high risk for HCC. However, AFP sensitivity for clinical HCC diagnosis is confined to 65%, with a preclinical predictive value below 40% (Liu et al. 2020; Sun et al. 2018). Therefore, it is of great significance to investigate novel, robust, and reliable biomarkers for early diagnosis and treatment of HCC.

Metabolomics has rapidly advanced in recent years, offering a valuable tool for tumor biomarker discovery, understanding pathogenesis, and tailoring personalized therapies. It involves a comprehensive analysis of cellular and organismal metabolites, unveiling aberrations in metabolic pathways, allowing a systemic overview of the organism’s physiology in disease and health (Chen et al. 2023; Debes et al. 2021; Liu et al. 2014; Luo et al. 2018; Zhang et al. 2012). The liver, a crucial organ, performs diverse metabolic functions essential for maintaining lipid and glucose homeostasis, as well as energy metabolism (Han et al. 2016). Since hepatocellular carcinoma is a complex molecular pathological process, it is characterized by intricate alterations in the genome, transcriptome, and metabolome levels (Beyoglu and Idle 2013). Therefore, metabolomics-based biomarkers are expected to be valuable tools for the early detection of HCC.

**Fig. 1:**
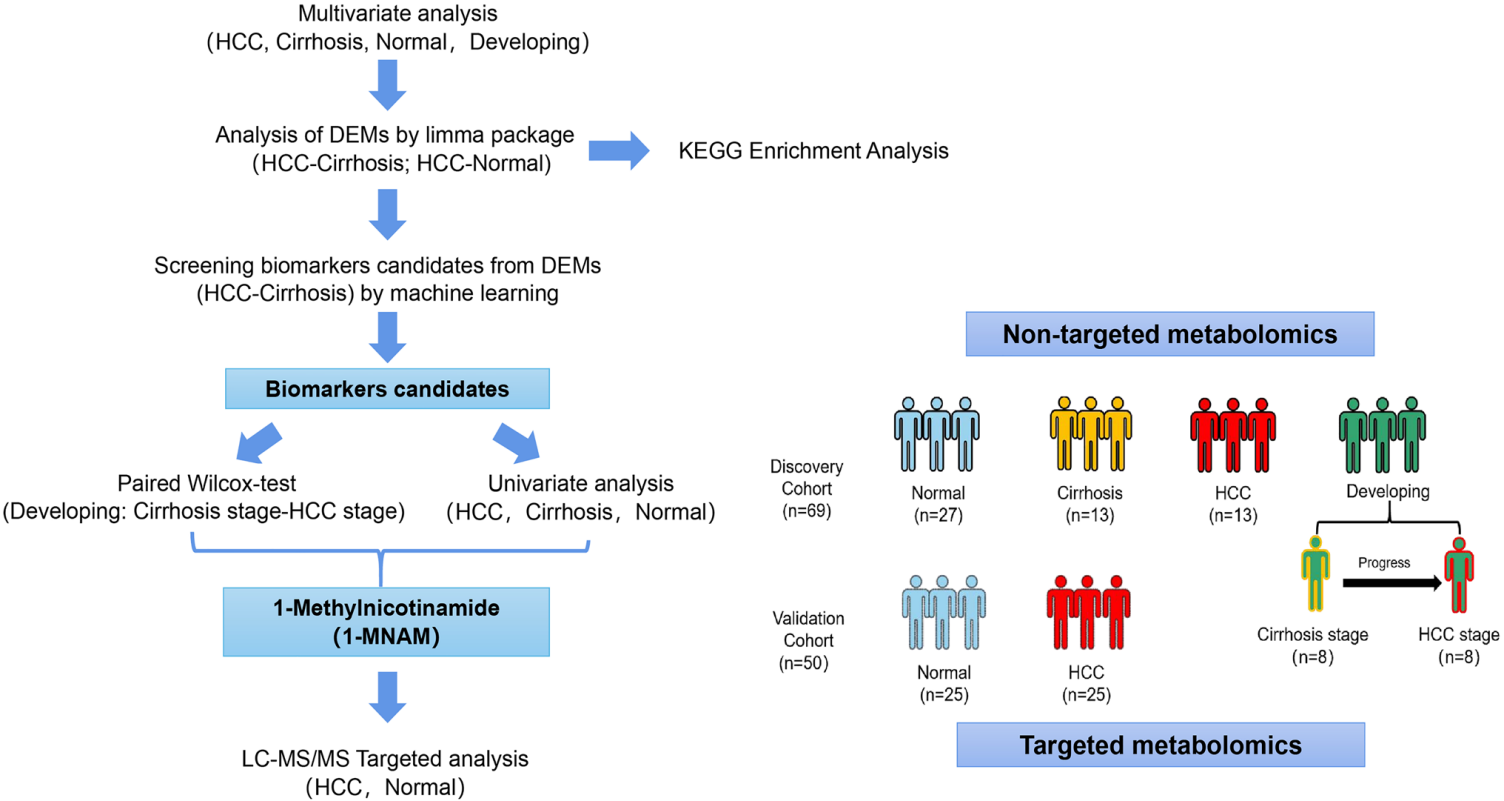
Overview of the study

In this study, we observed changes in metabolic profiles among patients progressing from cirrhosis to HCC following HBV infection. Figure 1 depicts the study design, which aims to identify serum biomarkers for early prediction of HCC. We explored the potential metabolic biomarkers with untargeted metabolomics techniques. Machine learning algorithms were used to identify key metabolic biomarkers and quantitative analysis in another independent cohort was constructed for validation. Our results indicate that 1-MNAM is the most promising metabolite for accurately differentiating HCC patients from those at risk. Additionally, decreased levels of 1-MNAM may serve as a sign of tumorigenesis in cirrhotic patients.

## Materials and methods

### Participants and sample preparation

The discovery cohort involved 69 participants, comprising healthy volunteers (Normal), liver cirrhosis patients with hepatitis B infection (Cirrhosis), hepatocellular carcinoma patients with chronic hepatitis B infection (HCC), and cirrhosis patients who developed cancer in 6–14 months (Developing) (Fig. 1). Blood samples were collected between February 2022 and December 2022. In the validation stage, 50 individual serums were collected (25 healthy subjects and 25 very early and early-stage HCC patients in Barcelona Clinic Liver Cancer stages 0 and A) between September 2022 and December 2022. Patients were clinically diagnosed with HBV-associated cirrhosis according to the “guideline of prevention and treatment for chronic hepatitis B in 2010” (Chinese Society of Hepatology 2011). The patient shall be diagnosed with chronic HBV infection if he or she had a previous history of hepatitis B or HBsAg positive for more than 6 months, and is presently HBsAg positive and (or) HBV DNA positive. Cirrhosis is diagnosed by imaging, biochemical, or hematological examinations showing dysfunction of hepatocytes or evidence of portal hypertension or histologically confirmed cirrhosis. HCC was diagnosed according to the “standards for the diagnosis and treatment of primary liver cancer” (China 2012). Clinical diagnosis of HCC can be established if the patient matches the following criteria: (1) if the diameter of the tumor is less than 2 cm, at least two of the four imaging tests, including dynamic enhanced MRI, dynamic enhanced CT, ultrasonography, or Gd-EOB-DTPA enhanced MRI, had characteristic HCC features. (2) If the tumor’s diameter is over 2 cm, at least one of the above four imaging tests shows characteristic HCC features. (3) If AFP is elevated (particularly the persistent elevation of AFP), at least one of the above four imaging tests shows characteristic HCC features. (4) Pathologic findings support the diagnosis of HCC. Healthy volunteers with a history of liver disease, systemic diseases, acute illnesses, or other malignancies were excluded. The baseline characteristics of participants are summarized in Table 1. Samples for both the discovery and validation phases were obtained from Ningbo No. 2 Hospital. The ethical approval was received, and all participants provided written informed consent.

**Table 1:** Diagnostic performances of serum metabolites from the discovery cohort (1-MNAM, AFP, CA19-9, CEA, CA125) in predicting HCC from normal people or cirrhosis patients

Subgroup	1-MNAM	AFP	CA19-9	CEA	CA125
HCC versus normal					
AUC	0.993	0.731	0.670	0.663	0.580
*T* tests	0.029	0.067	0.199	0.172	0.661
Log_2_ FC	−1.553	4.767	−0.626	−0.520	−0.241
HCC versus cirrhosis					
AUC	0.822	0.633	0.622	0.556	0.567
*T* tests	0.027	0.332	0.393	0.867	0.738
Log_2_ FC	1.049	−3.438	0.577	0.143	0.403

*1-MNAM* 1-Methylnicotinamide, *CA19-9* carbohydrate antigen 199, *CA-125* carbohydrate antigen 125, *AFP* alpha-fetoprotein, *CEA* carcinoembryonic antigen

Serum samples were collected in the morning after an overnight fast, processed, and stored at −80 °C before analysis. Sample preparation involved dilution with pre-cooled methanol, followed by vortexing and drying under nitrogen (Dunn et al. 2011).

### Untargeted metabolomics and measurement of metabolites

The experiments employed UHPLC (1290 Infinity LC, Agilent Technologies) coupled with a quadrupole time-of-flight mass spectrometer (AB Sciex Triple TOF 6600). HILIC separation was performed utilizing a 2.1 mm × 100 mm ACQUIY UPLC BEH 1.7 µm column (Waters, Ireland) in both positive and negative modes. The mobile phases consisted of phase A (25 mM ammonium acetate and 25 mM ammonium hydroxide in water) and phase B (25 mM ammonium acetate and 25 mM ammonium hydroxide in acetonitrile). RPLC separation utilized a 2.1 mm × 100 mm ACQUIY UPLC HSS T3 1.8 µm column (Waters, Ireland). In ESI positive mode, mobile phase A consisted of water with 0.1% formic acid, and mobile phase B comprised acetonitrile with 0.1% formic acid. In ESI negative mode, mobile phase A comprised 0.5 mM ammonium fluoride in water, and mobile phase B consisted of acetonitrile. Column temperatures were maintained at a constant 35℃. Each sample was injected with a 2 µL aliquot, and the scan range extended from 60 to 1000 *m*/*z*. Acquired mass data were imported into XCMS for peak detection and alignment, retaining only variables with more than 50% of non-zero measurement values across multiple groups. Missing values were substituted with half of the minimum abundance observed among all samples. Compound identification of metabolites was achieved by comparing the accuracy of *m*/*z* values (<25 ppm) and MS/MS spectra with an in-house database containing authentic standards.

### Targeted metabolomics and measurement of biomarker

Targeted metabolomics analysis was conducted using the Waters Acquity UPLC system. The mobile phases and UPLC gradient elution program mirrored those used in the untargeted metabolomics analysis with a UPLC BEH C18 column (2.1*50 mm, 1.7 μm). The autosampler and column compartment temperatures were set at 4 and 40℃, respectively. The MRM scan mode was employed for quantitative analysis. Data acquisition and processing were carried out using MassLynx 4.1 software (Milford, MA, USA).

### Differential expression analysis of metabolites

A supervised model of Partial Least Squares Discriminant Analysis (PLS-DA) with unit variance scaling was utilized to evaluate the overall changes in the metabolomes of the groups and to monitor the stability of the study. This work was done with SIMCA-P 14.0 software (Umetrics AB, Umeå, Sweden). Variance analysis of samples using the limma package of R software, with parameters |Log_2_ fold change| > 0.6 and *p* value <0.05 as screening criteria for DEMs. The expression data of these DEMs were visually represented using both a volcano plot and a heat map plot.

### Pathway analysis of DEMs

We utilized the web-based platform MetaboAnalyst (https://www.metaboanalyst.ca) to perform pathway enrichment analysis for DEMs, utilizing data from the Kyoto Encyclopedia of Genes and Genomes (KEGG, http://www.genome.jp/kegg). The significance threshold for pathway enrichment analysis was set at *p* < 0.05.

### Identification of disease signature metabolites

The machine learning algorithms Least absolute shrinkage and selection operator (LASSO) and random forest classifier (RF) were used to screen for significant biomarkers from potential metabolites. All these analyses were performed using the R package (randomforest and glmnet). Univariate analyses were conducted to detect biomarker concentrations among the Normal, Cirrhosis, and HCC groups, mitigating false positive errors inherent in multivariate statistics. Finally, the performance of selected biomarkers was evaluated by Binary logistic regression based on the area under the receiver operating characteristic curve (ROC).

### Correlation analysis for biomarker and liver function indexes

Correlation analysis between the given biomarker and liver function indexes was conducted using the Pearson correlation coefficient (r).

### Expression analysis of NNMT based on public database

TCGAplot R package (v4.0.0) (Liao and Wang 2023) was used to download the TPM matrix and clinical information for the 33 types of cancers in the TCGA database. Pan-cancer expression analysis for unpaired tumor-normal box plots using pan_boxplot functions of TCGAplot. To visually present the results, the “ggstatsplot” and “ggplot2” packages were utilized. Employ the Kaplan–Meier method for survival analysis to examine the relationship between overall survival time and NNMT expression. Determine the statistical significance of overall survival by utilizing the Log-Rank test.

## Results

### Metabolic profiling of serum

**Fig. 2:**
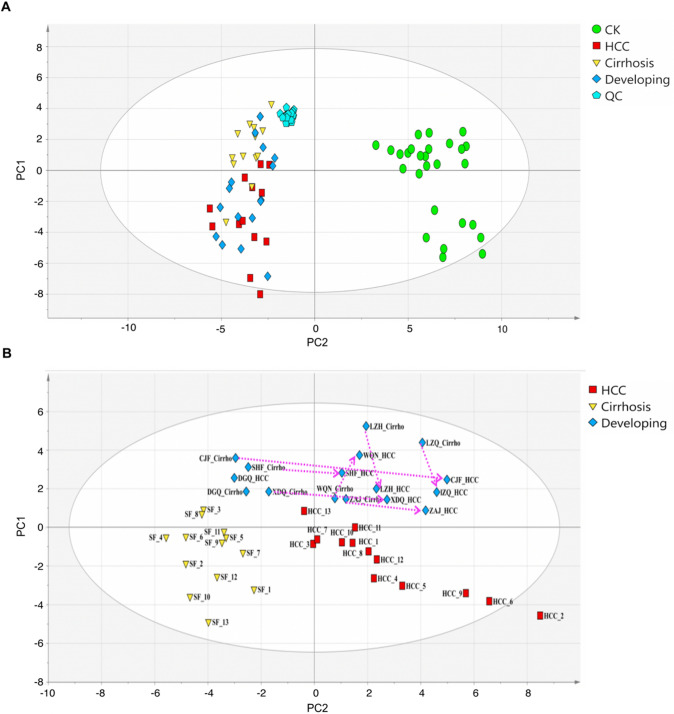
Metabolic profiling of discovery cohort. **A** Principal component analysis (PCA) score plots of serum samples collected from the discovery set. **B** Partial least squares discriminant analysis (PLS-DA) based on Cirrhosis, Developing, and HCC groups. The score plot reflects the tendency of the serum metabolic fluctuations during disease progression. 7 patients from developing groups in the HCC stage (abbreviated name_HCC) were located on the right side of the cirrhosis stage (abbreviated name_Cirrho), which were marked by a dotted line with an arrowhead

Using the UPLC-TOF-HDMS system, 17,758 variables were detected, including 9325 in positive ion mode and 8433 in negative ion mode from 69 participants in the discovery set. 115 metabolites were identified based on the in-house database established with available authentic standards and online databases (Metlin and ChemSpider). In the PLS-DA score plot (Fig. 2A), the QC samples cluster tightly together, which indicates that the present analysis was stable. Furthermore, the clear separation among the Normal, Cirrhosis, and HCC groups revealed significant systematic metabolic differences.

During the follow-up period, eight subjects from the developing group experienced progression from hepatitis B-induced cirrhosis to HCC. The changes in their plasma metabolome were analyzed to identify biomarkers associated with the development of HCC, and the tendency was demonstrated by the red arrows pointing from the cirrhosis stage to the HCC stage for each patient. The PLS-DA plot demonstrated that in seven out of eight subjects, the metabolic profile changes were consistent with the transition from cirrhosis to HCC (Fig. 2B). Specifically, the cirrhosis stage was closer to the third quadrant, where the Cirrhosis group was located, while the HCC stage was closer to the fourth quadrant, where the HCC group was clustered. This result suggests that blood metabolic profiles change significantly during cirrhosis progresses to HCC.

### Identification of aberrant metabolites and pathway enrichment

**Fig. 3:**
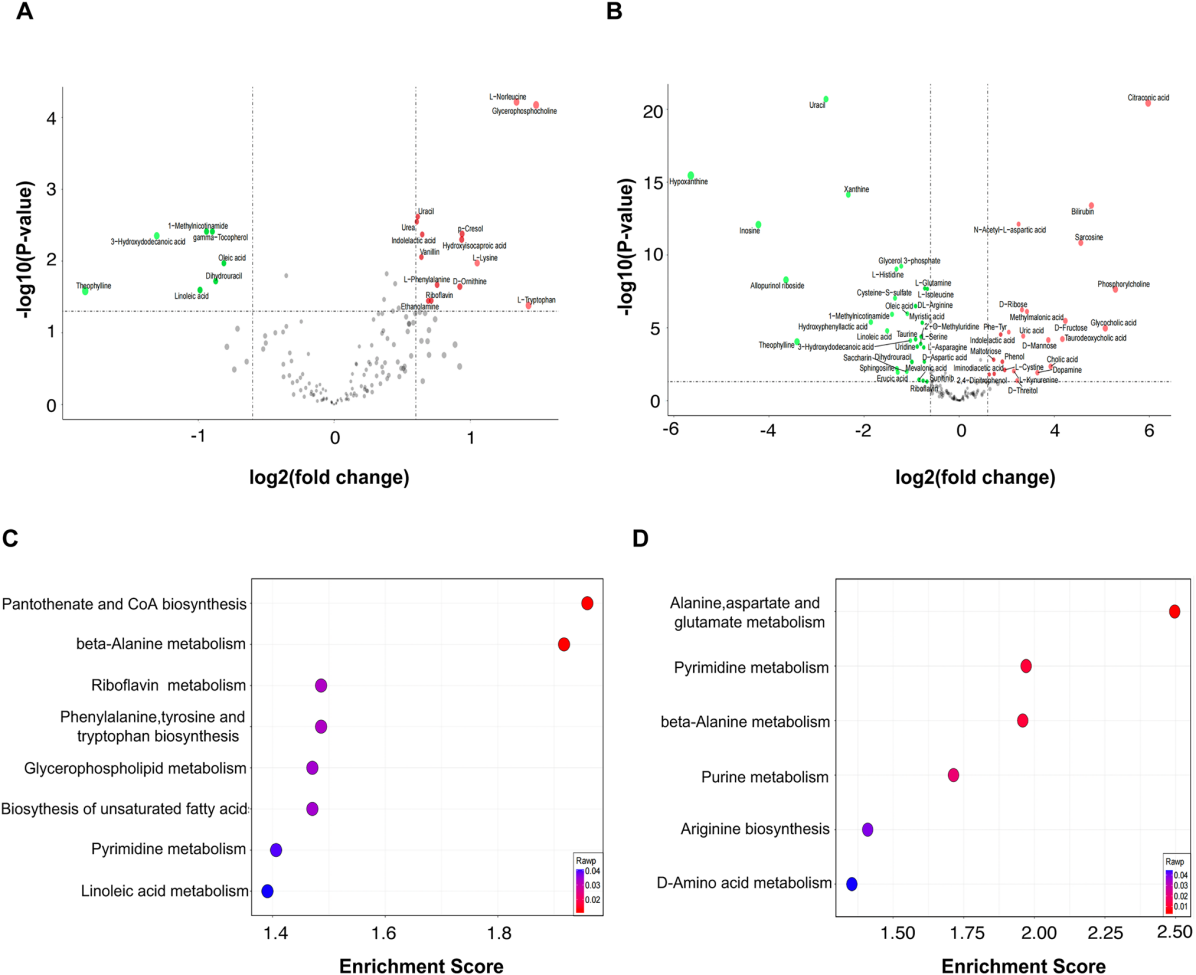
Identification of differentially expressed metabolites and KEGG enrichment. **A** Volcano plot of DEMs between Cirrhosis and HCC group. **B** Volcano plot of DEMs between Normal and HCC group. **C** Dot graph showing the significantly enriched KEGG pathway according to DEMs between Cirrhosis and HCC group. **D** Dot graph showing the significantly enriched KEGG pathway according to DEMs between Normal and HCC groups

Differential analysis was employed to identify metabolites with abnormal changes (DEMs) between the Cirrhosis and HCC groups, as well as between the Normal and the HCC groups. A total of 14 upregulated and 7 downregulated metabolites were identified when comparing patients with cirrhosis and liver cancer. Similarly, 23 upregulated and 31 downregulated metabolites were discovered in liver cancer patients compared to healthy individuals. Volcano plots illustrating the DEMs are depicted in Fig. 3A and B, with the detailed information provided in Tables S1. and S2

Additionally, a KEGG enrichment analysis was conducted to explore potential regulatory pathways of DEMs. Enrichment analysis revealed significant enrichment in pathways between the Cirrhosis and HCC groups, including “pantothenic acid and CoA biosynthesis”, “β-alanine metabolism”, “phenylalanine, tyrosine, and tryptophan biosynthesis”, “riboflavin metabolism”, “unsaturated fatty acid biosynthesis”, “glycerophospholipid metabolism”, “pyrimidine metabolism”, and “linoleic acid metabolism” (Fig. 3C). DEMs from Normal and HCC comparison were mainly enriched in “alanine, aspartate, and glutamate metabolism”, “pyrimidine metabolism”, “β-alanine metabolism” and “purine metabolism” (Fig. 3D).

### Identifying potential biomarkers for the progression from cirrhosis to HCC

**Fig. 4:**
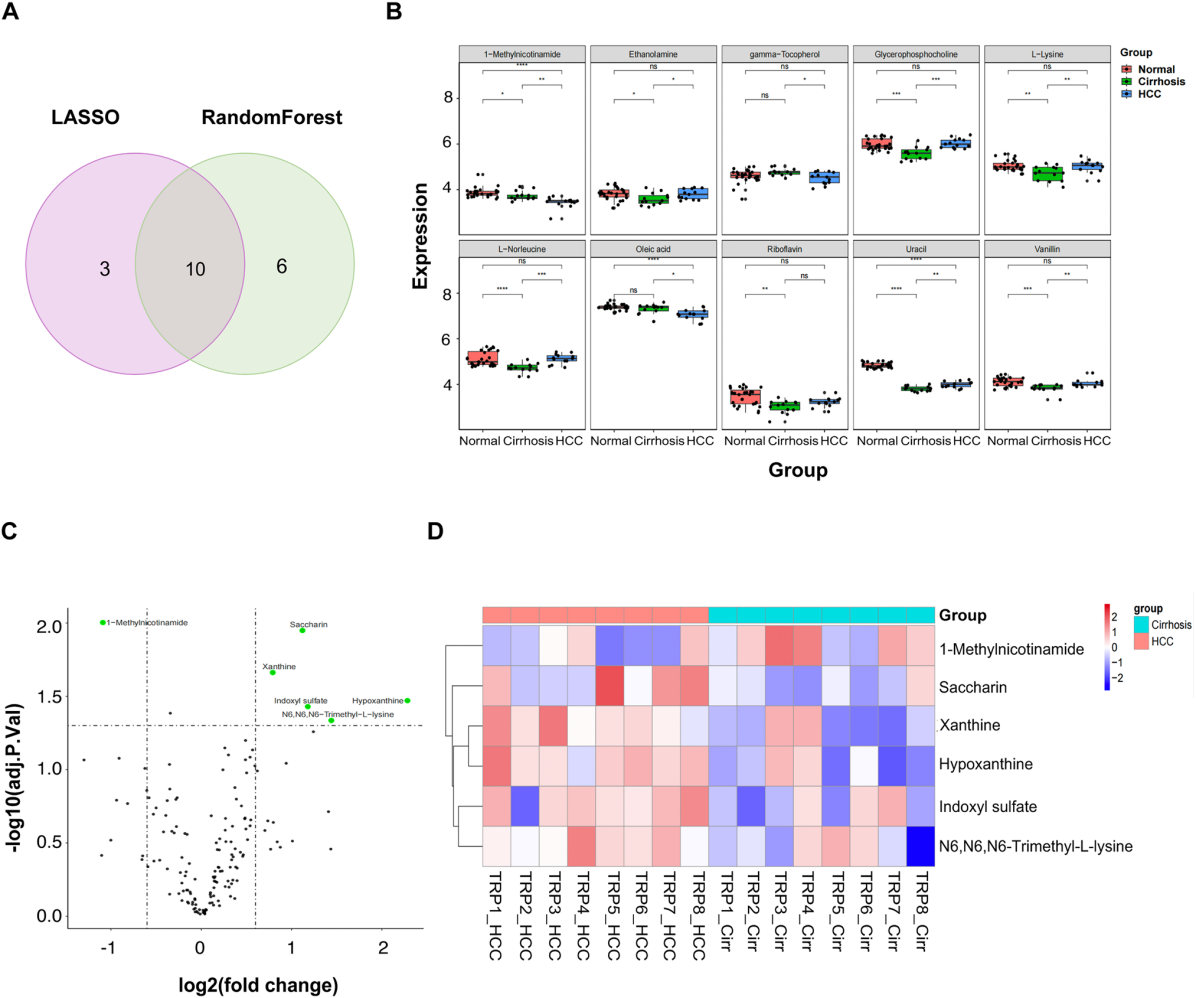
Identifying potential biomarkers for the progression from cirrhosis to HCC. **A** Venn plot of the overlapping genes identified through the two machine algorithms. **B** Distribution of ten potential biomarkers in Normal, Cirrhosis, and HCC groups. **C** Volcano plots showing DEMs (points in green with label) between the cirrhosis stage and HCC stage of 8 patients in the Developing group. **D** Heatmap of altered metabolites between the cirrhosis stage and HCC stage of 8 patients in the Developing group

LASSO regression and random forest models were utilized to narrow down the DEMs between the Cirrhosis and HCC groups, leading to the identification of 10 DEMs as potential markers for distinguishing HCC cases from cirrhosis patients (Fig. 4A). Subsequently, these potential markers underwent further shortlisting through univariate analysis. Notably, only 1-MNAM consistently exhibited a decrease across the Normal, Cirrhosis, and HCC groups (Fig. 4B). In alignment with the previous findings, a comparison of the metabolic profiles between the cirrhotic and HCC stages within the Developing group revealed that 1-MNAM was the most significantly decreased compound during the transition from cirrhosis to HCC (Fig. 4C, D). Additionally, we compared the efficacy of 1-MNAM using the discovery set and evaluated its performance by assessing the area under the receiver operating characteristic curve (AUC). For HCC diagnosis, the AUC values were 0.992 and 0.822 for the Normal and Cirrhosis groups, respectively, surpassing those of AFP, CA19-9, CEA, and CA12-5 (Table 1).

### Validation of 1-MNAM for HCC diagnostic performance

**Fig. 5:**
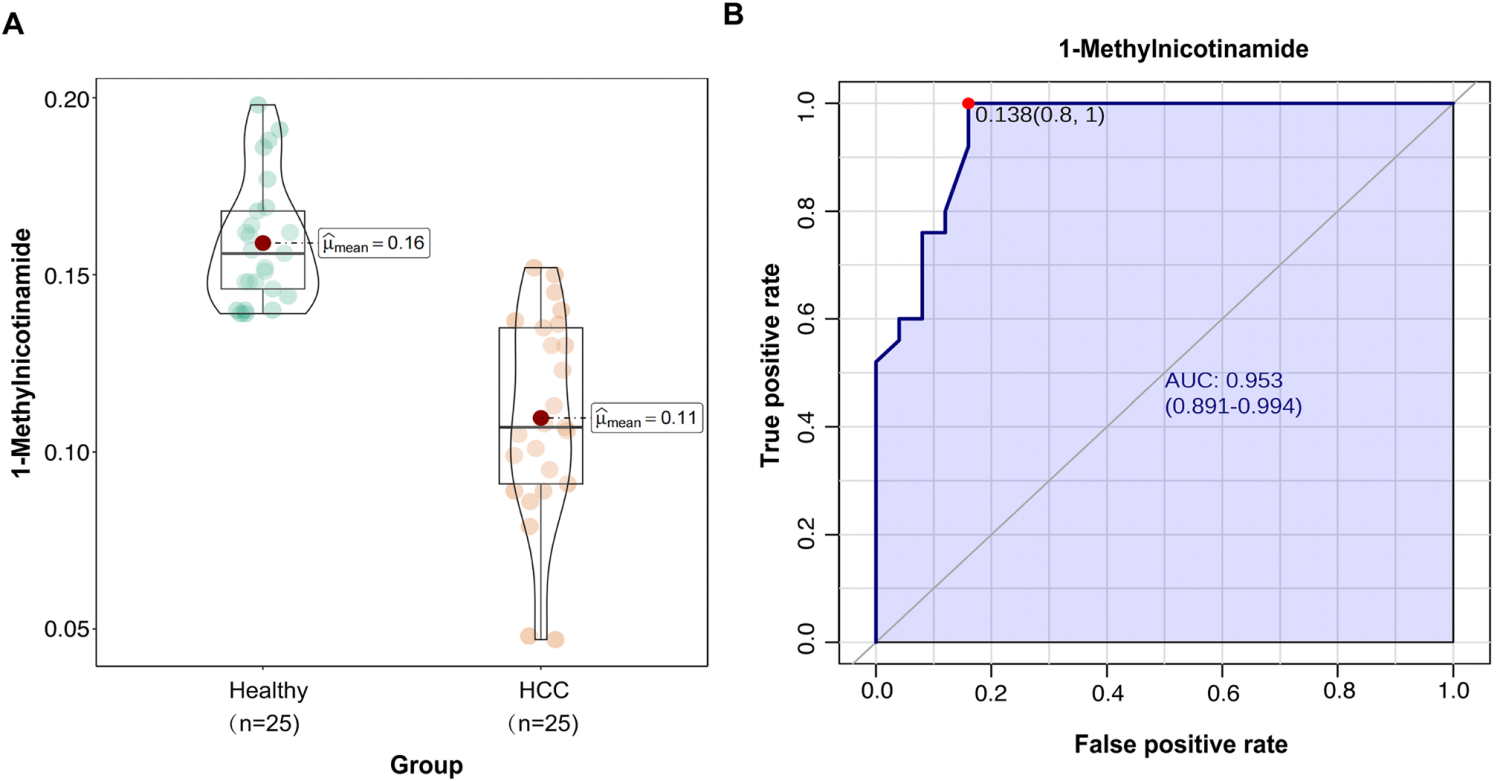
Validation of 1-Methylnicotinamide for HCC diagnostic performance. **A** Violin plot of 1-Methylnicotinamide levels within Normal and HCC groups from external validation cohort. **B** Receiver operating characteristics and area under the curve (AUC) of 1-Methylnicotinamide to differentiate HCC cases from normal control in the validation cohort

To further validation of diagnostic performance, we measured the precise abundance of 1-MNAM using targeted mass spectrometric assays in an additional independent cohort comprising 25 healthy individuals and 25 HCC patients with HBV infection. The baseline characteristics are shown in Table 2. Consistent with the above findings, HCC patients exhibited significantly lower levels of 1-MNAM compared to healthy controls. ROC analyses further confirmed the high diagnostic performance of 1-MNAM, with an AUC of 0.954 (Fig. 5).

**Table 2:** Baseline characteristics of participants in the discovery and validation sets

	Discovery set (*n* = 69)				*p* value	Validation set (*n* = 50)		*p* value
Characteristic	Normal	Cirrhosis	HCC	Developing		Normal	HCC	
*n*	27	13	13	16		25	25	
Age (years)	51.44 ± 8.47	58.15 ± 7.16	57.71 ± 8.57	53.33 ± 5.84	0.647	46.72 ± 6.26	54.28 ± 8.07	0.478
Sex (male/female)	17/10	7/6	12/2	5/3	0.297	16/9	21/4	0.315
AFP (ng/mL)	1.26 ± 1.35	1.64 ± 2.25	10,455.9 ± 20,851.52	17.06 ± 21.51	0.008	–	10,455.9 ± 20,851.52	0.02
CA19-9 (ng/ml)	12.15 ± 8.51	11.82 ± 10.06	7.88 ± 9.69	17.04 ± 11.46	0.155	–	16.58 ± 25.77	0.42
CA-125 (ng/ml)	2.03 ± 2.45	2.2 ± 2.62	0.9 ± 1.64	7.79 ± 11.7	0.017	–	38.18 ± 104.85	0.098
CEA (ng/ml)	0.28 ± 0.41	0.1 ± 0.58	0.1 ± 0.32	0.7 ± 0.97	0.026	–	1.64 ± 3.09	0.039
GGT (U/L)	32.5 ± 30.1	64.4 ± 35.0	76.4 ± 87.5	58.9 ± 42.4	0.052	–	189 ± 247	0.005
ALT (U/L)	23.5 ± 14.4	30.4 ± 12.8	40.7 ± 24.6	31.6 ± 10.2	0.027	–	32.6 ± 21.9	0.09
ChE (U/L)	8762 ± 1643	6189 ± 2331	6242 ± 2133	5320 ± 1892	<0.001	–	5122 ± 2753	<0.001
ALP (U/L)	67.7 ± 17.2	105 ± 66.2	97.6 ± 54.6	101 ± 36.7	0.02	–	178 ± 132	<0.001
AST (U/L)	19.4 ± 7.03	38.5 ± 10.9	32.8 ± 18.2	36.4 ± 7.99	<0.001	–	53.4 ± 43.4	0.001
Lpa (U/L)	131 ± 166	159 ± 165	90.5 ± 72.3	77.4 ± 33.4	0.405	–	216 ± 258	0.176
TBIL (μmol/L)	12.0 ± 3.56	16.1 ± 3.36	13.1 ± 4.66	13.3 ± 8.67	0.242	–	16.0 ± 8.35	0.04
DBIL (μmol/L)	4.03 ± 1.14	6.01 ± 2.48	5.15 ± 1.62	6.40 ± 6.14	0.122	–	7.08 ± 3.88	0.345
TBA (μmol/L)	4.20 ± 2.61	29.4 ± 42.8	16.5 ± 18.9	29.5 ± 23.6	0.002	–	22.5 ± 28.0	0.004
AST:ALT	1.00 ± 0.53	1.32 ± 0.35	0.88 ± 0.40	1.22 ± 0.35	0.077	–	1.70 ± 0.82	0.001

*GGT* γ-glutamyl transferase, *ALT* alanine aminotransferase, *ChE* cholinesterase, *ALP* alkaline phosphatase, *AST* aspartate aminotransferase, *TBIL* total bilirubin, *Lpa* lipoprotein *a*, *DBIL* direct bilirubin, *TBA* total bile acid

### Correlation analysis between 1-MNAM and Clinical characteristic

**Fig. 6:**
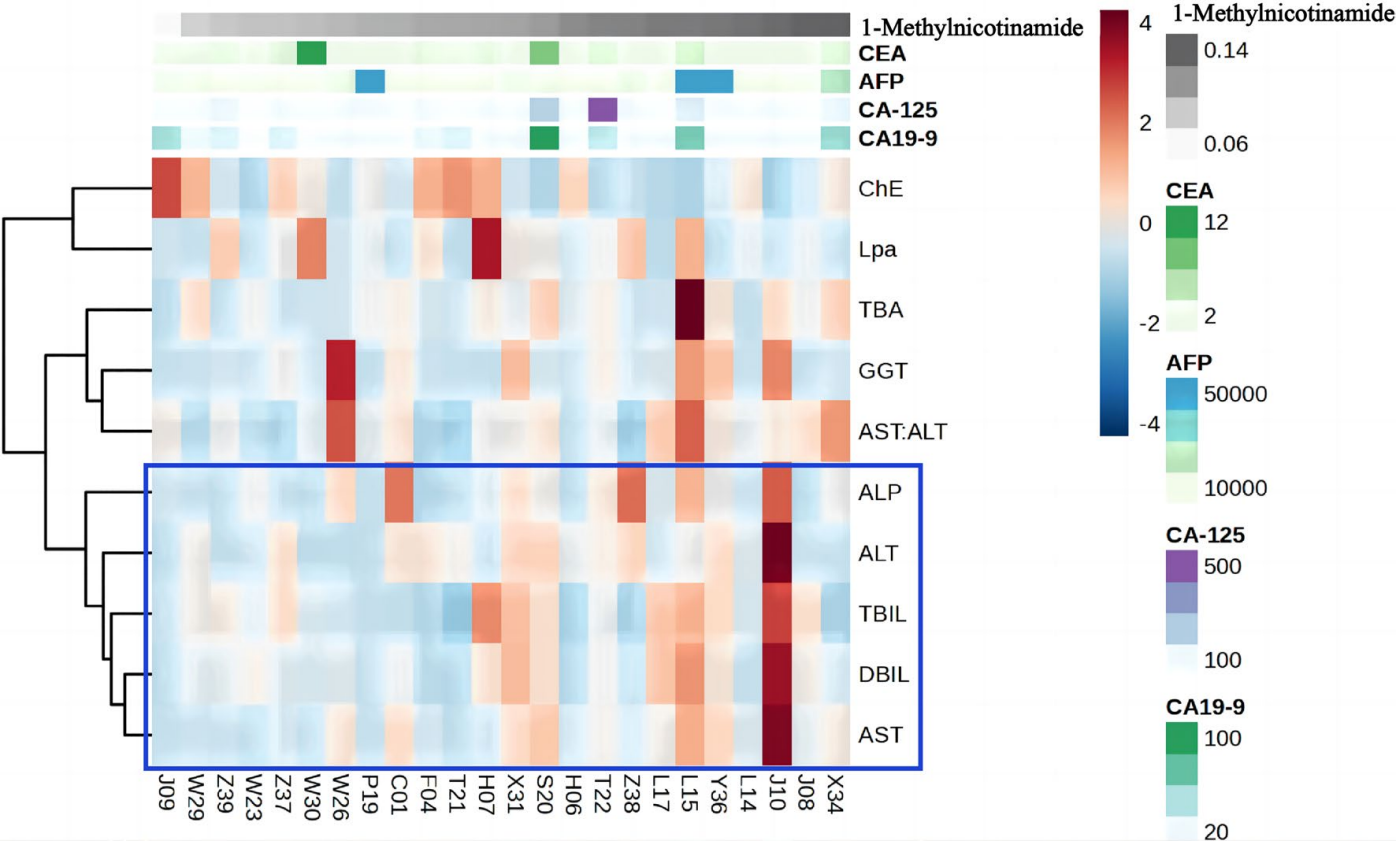
Heatmap shows correlation value among all groups. The concentration of biomarkers is color-coded by row sidebars, and the dendrogram is located on the left of the color-coded sidebars. Each row corresponds to liver enzymes and function indexes, and each column corresponds to HCC patients from the validation cohort. The colors are scaled by row; red and blue indicate 4 and −4, respectively

Correlation analysis examined the relationship between 1-MNAM and liver function indexes among the HCC patients in the validation cohort. The levels of ALP, ALT, AST, TBIL, and DBIL were positively associated with 1-MNAM. In contrast, ChE was negatively associated with 1-MNAM (Fig. 6). Furthermore, compared to widely utilized blood tumor markers (such as AFP, CA19-9, CEA, and CA12-5), 1-MNAM demonstrated a more robust association with hepatic functional impairment (Fig. 6).

## Discussion

In this study, we observed a consistent trend in the metabolic profiles of the majority of Developing patients transitioning from the cirrhosis stage to the early HCC stage, gradually aligning with the metabolic state of HCC patients. Thus, we inferred that alterations in blood metabolic profiles were closely associated with the progression from cirrhosis to HCC and could potentially help in diagnosing early-stage HCC patients from those with cirrhosis. We employed an untargeted metabolomics approach to investigate the serological profiles of patients with liver cirrhosis and early HCC associated with chronic hepatitis B virus infection, along with paired comparisons of serum metabolites between cirrhotic and HCC stages in patients with hepatitis B-induced hepatocellular carcinoma. Through integration with machine learning algorithms and multivariate statistical analyses, we identified 1-MNAM as a metabolic diagnostic biomarker associated with the progression from hepatitis B cirrhosis to HCC and validated its diagnostic efficacy in the targeted metabolome. Furthermore, 1-MNAM demonstrated superior capability in distinguishing HCC from the high-risk population (cirrhosis) compared to traditional blood markers (such as AFP, CA19-9, and CA125).

Untargeted metabolomics analysis has been applied as an important technique for resolving metabolic changes in complex diseases, including cancer, diabetes, and cardiovascular disease. Statistical analysis showed metabolic differences between Cirrhotic and HCC samples, with 21 dysregulated metabolites identified. Similarly, 54 dysregulated metabolites were identified in the HCC group compared to the Normal group. KEGG analysis revealed that “β-alanine metabolism” and “pyrimidine metabolism” were dysregulated in both two comparisons. β-alanine is an important metabolite in humans and is involved in a variety of metabolic pathways, including gluconeogenesis and fatty acid synthesis (Metallo et al. 2011; Owen et al. 2002). Since the liver is one of the major organs for β-alanine metabolism, abnormal β-alanine metabolism may reflect abnormal liver function. Studies have shown that abnormal alanine metabolism may be associated with hepatic inflammation, fibrosis, and tumorigenesis. During the development of liver diseases such as chronic hepatitis and cirrhosis, abnormal β-alanine metabolism may affect the growth and proliferation of hepatocytes, thus promoting the development of HCC (Casadei-Gardini et al. 2020; Gaggini et al. 2018). Pyrimidine metabolism (PyMet) is a branch of nucleotide metabolism that plays an important role in maintaining cellular nucleic acid synthesis and metabolic homeostasis (Loffler et al. 2005). Dysregulation of pyrimidine metabolism may lead to various pathologic features of cancer, including chemoresistance and epithelial-mesenchymal transition (Ramesh et al. 2024). Moreover, previous studies have shown that dysfunctional pyrimidine metabolism is strongly associated with cancer progression, and several drugs targeting pyrimidine metabolism have been approved for the treatment of multiple types of cancer (Wang et al. 2021).

Machine learning plays a crucial role in the research of clinical disease markers. Compared with traditional methods of marker screening, integrated machine learning methods based on multiple feature selection algorithms can screen potential biomarkers with higher sensitivity, accuracy, and stability, and can construct efficient and stable diagnostic models. In this study, we screened the characteristic metabolism-related biomarker 1-MNAM using two machine learning methods, LASSO and RF. As cirrhosis transitioned to HCC, we found a consistent decline in 1-MNAM levels. Furthermore, patients with cirrhosis and HCC exhibited significantly reduced 1-MNAM concentrations compared to healthy individuals. Consequently, we postulated a potential association between 1-MNAM and HCC development, suggesting that the declining trend in 1-MNAM levels might signal pre-cancer progression.1-MNAM, primarily synthesized in the liver by Nicotinamide *N*-methyltransferase (NNMT), is naturally occurring in human plasma and exhibits diverse biological activities, notably anti-inflammatory and antithrombotic effects (Chlopicki et al. 2007; Jakubowski et al. 2016). NNMT catalyzes the transfer of reactive methyl groups from *S*-adenosylmethionine (SAM) to nicotinamide (NAM), resulting in the production of 1-MNAM (Wang et al. 2022) (Fig. S1-A). Our preliminary experimental findings indicated no significant difference in the levels of NAM and SAM between liver cancer patients and the healthy population. Considering NNMT’s pivotal role in 1-MNAM metabolism, we hypothesized that the decreased levels of 1-MNAM in HCC could be linked to the dysregulated expression of NNMT.

Many studies have reported that NNMT was associated with various types of cancer, including primary glioblastoma (Jung et al. 2017), bladder (Pozzi et al. 2018), ovarian (Harmankaya et al. 2021), and colorectal cancers (Song et al. 2020). Analysis of pan-cancer expression patterns in the TCGA dataset revealed a substantial decrease in NNMT expression in HCC compared with paraneoplastic tissues (Fig. S1-B). Under physiological conditions, NNMT expression is typically high in the liver, but its protein levels are suppressed in HCC, particularly in the early stage (Zhang et al. 2021). Additionally, analysis of TCGA and CPTAC databases revealed significantly reduced mRNA and protein expression levels of NNMT in HCC tissues compared to paraneoplastic tissues (Fig. S1C–D), and low NNMT expression correlated with poor prognosis in HCC patients (Fig. S1-E). Consistent with our findings, previous studies have shown that Acox2^−/−^ mice, exhibiting clinical signs of NAFLD, displayed significantly reduced blood and liver levels of 1-MNAM and NNMT expression (Zhang et al. 2021). Additionally, 28.57% of these mice developed HCC due to steatosis, inflammation, and fibrosis. Furthermore, investigations in NASH mice with NNMT knockout (NNMT-LKO) revealed notable reductions in total cholesterol levels, low-density lipoprotein levels, liver weight, liver inflammation, and fibrosis levels. Further analysis revealed that the levels of liver damage markers in the serum of NNMT-LKO mice were significantly decreased (Li et al. 2022). These findings imply a potential association between reduced 1-MNAM levels in HCC and NNMT, and the suppression of NNMT expression could be linked to the liver steatosis-inflammation-cancer axis. However, the precise mechanisms underlying their roles in HCC remain unclear and need further exploration. Previous studies have found that NNMT and Sirtuin 1 (Sirt1) overlap in liver physiology and pathophysiology (Ding et al. 2017; Hao et al. 2014; Qiu et al. 2021). NNMT regulates glucose, lipid, and cholesterol metabolism by stabilizing Sirt1 (Hong et al. 2015). Moreover, it has been shown that the downregulation of NNMT contributes to the survival of HCC cells by enhancing autophagy under nutrient starvation, thus autophagy inhibitor therapy may provide a possible therapeutic intervention for HCC by inhibiting NNMT (Roberti et al. 2021; Shin et al. 2018). In summary, NNMT may be one of the important drug target genes for liver metabolic diseases.

There are also some limitations to our study. Firstly, the diagnostic accuracy of 1-MNAM needs to be fully validated in large prospective studies and extensively verified through in vitro and in vivo experiments, considering the small number of enrolled patients in our study. Secondly, it is essential to conduct tests on HCC patients at different stages to comprehend the trends in 1-MNAM and NNMT levels throughout the progression from cirrhosis to HCC. Thirdly, the absence of follow-up, treatment, and therapeutic information in our study precluded us from examining the influence of selected biomarkers on treatment outcomes’ quality or evaluating their predictive performance.

## Conclusion

This study investigated changes in serum metabolic profiles in patients transitioning from cirrhosis to HCC during follow-up, and 1-Methylnicotinamide appeared to be associated with the progression of cirrhosis. Additionally, the concentration of 1-MNAM significantly decreased in HCC cases in both the discovery and validation cohorts, as determined by untargeted and targeted metabolomics analyses, respectively. The significant decrease of 1-MNAM in the blood is expected to indicate the progression of HBV-cirrhosis to HCC, and 1-MNAM shows promise as a potential marker for the early detection of HCC in high-risk patients.

### Supplementary Information

Below is the link to the electronic supplementary material.

Supplementary file1 (DOCX 955 KB)

## Data Availability

The original study data could be required from the corresponding author upon reasonable request.
