# Supplementary material for: Identification of 1-Methylnicotinamide as a specific biomarker for the progression of cirrhosis to hepatocellular carcinoma
Source: J Cancer Res Clin Oncol. 2024 Jun 18;150(6):310. doi: 10.1007/s00432-024-05848-6 (PMC11189347; doi:10.1007/s00432-024-05848-6)
Supplement: Supplementary file 1 — Supplementary file1 (DOCX 955 KB) [file 432_2024_5848_MOESM1_ESM.docx]

## Supplementary Materials

**Fig.S1** Pan-cancer analysis and expression analysis in HCC of NNMT. **A** NAM-methylation reaction catalyzed by NNMT(Wang et al., 2022). **B** Pan-cancer expression of NNMT. In the coordinate axis, red represents a high expression of NNMT in the tumor tissue, while blue represents a low expression of NNMT in the tumor tissue. *p<0.05, **p<0.01, ***p<0.001, ****p<0.0001. **C** mRNA expression of NNMT in paired samples from TCGA-LIHC cohort. **D** Protein expression of NNMT in paired samples from CPTAC database. **E** KM plot for the survival analysis of NNMT expression in HCC.


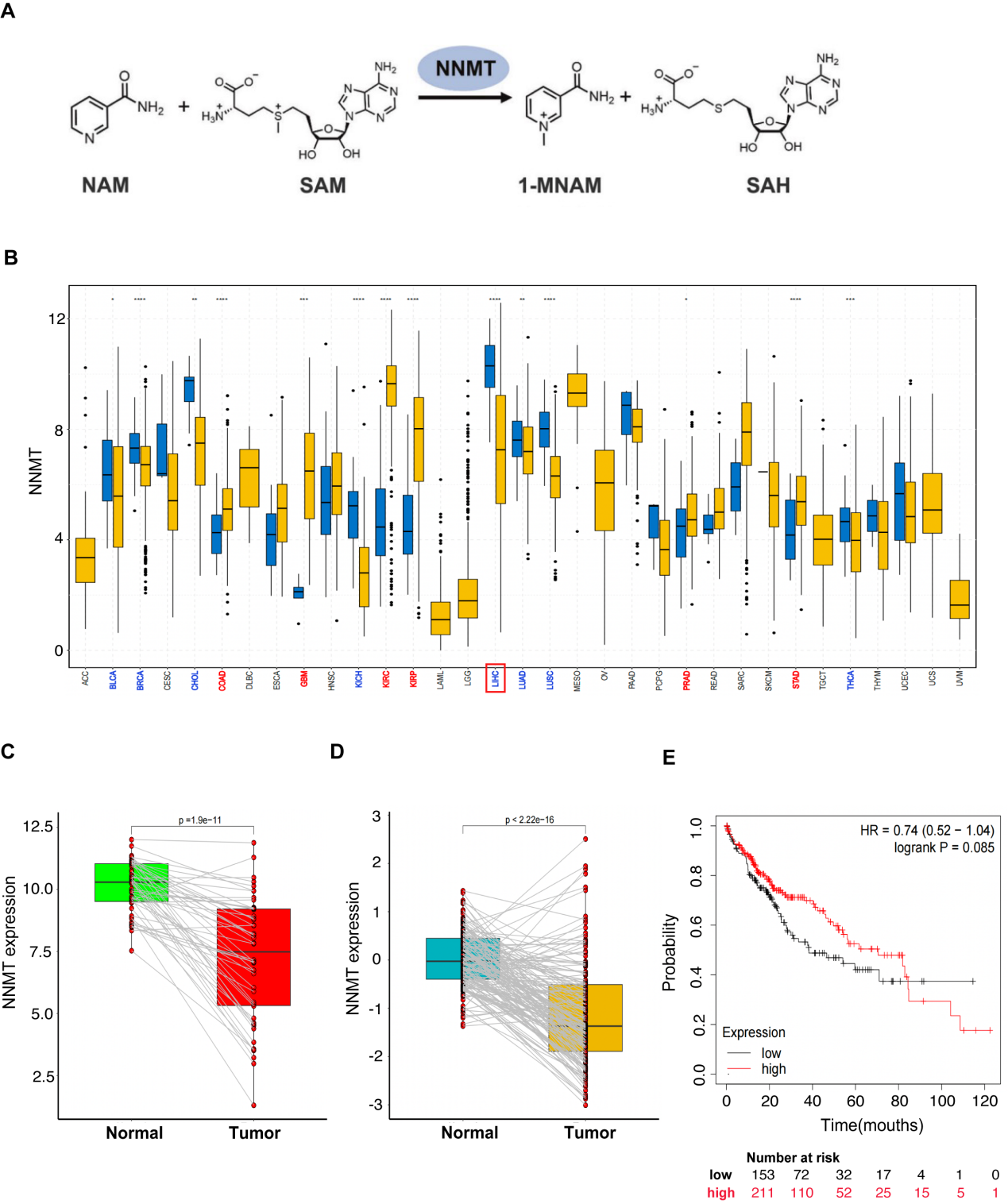


**Table S1** Significant differential metabolites (DEMs) identified between Cirrhosis and HCC group

| DEMs | logFC | Average Expression | *P* value | FDR |
| --- | --- | --- | --- | --- |
| L-Norleucine | 1.34E+00 | 1.63E+01 | 6.08E-05 | 5.12E-03 |
| Glycerophosphocholine | 1.48E+00 | 1.93E+01 | 6.69E-05 | 5.12E-03 |
| Uracil | 6.14E-01 | 1.30E+01 | 2.41E-03 | 7.56E-02 |
| Urea | 6.06E-01 | 1.70E+01 | 2.83E-03 | 7.56E-02 |
| 1-methylnicotinamide | -9.39E-01 | 1.19E+01 | 3.88E-03 | 7.56E-02 |
| gamma-Tocopherol | -8.96E-01 | 1.54E+01 | 3.88E-03 | 7.56E-02 |
| p-Cresol | 9.39E-01 | 1.40E+01 | 4.19E-03 | 7.56E-02 |
| Indolelactic acid | 6.46E-01 | 1.45E+01 | 4.26E-03 | 7.56E-02 |
| 3-Hydroxydodecanoic acid | -1.30E+00 | 1.62E+01 | 4.45E-03 | 7.56E-02 |
| Hydroxyisocaproic acid | 9.37E-01 | 1.59E+01 | 5.02E-03 | 7.68E-02 |
| Vanillin | 6.40E-01 | 1.31E+01 | 8.81E-03 | 1.23E-01 |
| L-Lysine | 1.05E+00 | 1.62E+01 | 1.07E-02 | 1.26E-01 |
| Oleic acid | -8.11E-01 | 2.39E+01 | 1.07E-02 | 1.26E-01 |
| Dihydrouracil | -8.71E-01 | 1.24E+01 | 1.91E-02 | 1.82E-01 |
| L-Phenylalanine | 7.56E-01 | 1.84E+01 | 2.15E-02 | 1.83E-01 |
| D-Ornithine | 9.23E-01 | 1.39E+01 | 2.27E-02 | 1.83E-01 |
| Linoleic acid | -9.86E-01 | 1.77E+01 | 2.54E-02 | 1.91E-01 |
| Theophylline | -1.83E+00 | 1.30E+01 | 2.62E-02 | 1.91E-01 |
| Riboflavin | 7.13E-01 | 1.04E+01 | 3.57E-02 | 2.29E-01 |
| Ethanolamine | 6.91E-01 | 1.23E+01 | 3.59E-02 | 2.29E-01 |
| L-Tryptophan | 1.43E+00 | 1.58E+01 | 4.16E-02 | 2.54E-01 |

**Table S2** Significant differential metabolites (DEMs) identified between Normal and HCC group

| DEMs | logFC | Average Expression | *P* value | FDR |
| --- | --- | --- | --- | --- |
| Uracil | -2.80E+00 | 1.52E+01 | 1.98E-21 | 2.84E-19 |
| Citraconic acid | 3.98E+00 | 1.34E+01 | 3.71E-21 | 2.84E-19 |
| Hypoxanthine | -5.64E+00 | 1.96E+01 | 3.51E-16 | 1.79E-14 |
| Xanthine | -2.33E+00 | 1.62E+01 | 7.23E-15 | 2.77E-13 |
| Bilirubin | 2.78E+00 | 1.42E+01 | 3.97E-14 | 1.21E-12 |
| N-Acetyl-L-aspartic acid | 1.25E+00 | 1.35E+01 | 7.59E-13 | 1.80E-11 |
| Inosine | -4.22E+00 | 1.43E+01 | 8.23E-13 | 1.80E-11 |
| Sarcosine | 2.56E+00 | 1.43E+01 | 1.46E-11 | 2.78E-10 |
| Glycerol 3-phosphate | -1.22E+00 | 1.07E+01 | 5.85E-10 | 9.95E-09 |
| L-Histidine | -1.32E+00 | 1.81E+01 | 9.23E-10 | 1.41E-08 |
| Allopurinol riboside | -3.64E+00 | 1.40E+01 | 5.10E-09 | 7.10E-08 |
| L-Glutamine | -7.23E-01 | 1.74E+01 | 1.96E-08 | 2.48E-07 |
| L-Isoleucine | -6.67E-01 | 1.35E+01 | 2.13E-08 | 2.48E-07 |
| Phosphorylcholine | 3.29E+00 | 1.16E+01 | 2.27E-08 | 2.48E-07 |
| Cysteine-S-sulfate | -1.35E+00 | 1.10E+01 | 9.22E-08 | 9.40E-07 |
| DL-Arginine | -9.17E-01 | 1.47E+01 | 3.25E-07 | 3.10E-06 |
| D-Ribose | 1.32E+00 | 1.46E+01 | 5.81E-07 | 5.23E-06 |
| Methylmalonic acid | 1.43E+00 | 1.50E+01 | 7.59E-07 | 6.45E-06 |
| Oleic acid | -1.09E+00 | 2.42E+01 | 1.08E-06 | 8.69E-06 |
| 1-methylnicotinamide | -1.41E+00 | 1.24E+01 | 1.18E-06 | 9.00E-06 |
| D-Fructose | 2.23E+00 | 1.42E+01 | 3.35E-06 | 2.44E-05 |
| Hydroxyphenyllactic acid | -1.86E+00 | 1.28E+01 | 4.05E-06 | 2.69E-05 |
| Myristic acid | -7.76E-01 | 1.82E+01 | 4.46E-06 | 2.84E-05 |
| Glycocholic acid | 3.07E+00 | 1.44E+01 | 1.07E-05 | 6.29E-05 |
| Linoleic acid | -1.51E+00 | 1.82E+01 | 1.61E-05 | 9.10E-05 |
| Phe-Tyr | 1.04E+00 | 1.07E+01 | 1.98E-05 | 1.08E-04 |
| Indolelactic acid | 8.74E-01 | 1.43E+01 | 2.87E-05 | 1.52E-04 |
| Uric acid | 1.35E+00 | 1.11E+01 | 3.68E-05 | 1.88E-04 |
| 2'-O-Methyluridine | -8.00E-01 | 1.34E+01 | 4.06E-05 | 2.00E-04 |
| Taurodeoxycholic acid | 2.17E+00 | 1.26E+01 | 5.90E-05 | 2.82E-04 |
| Taurine | -9.16E-01 | 1.87E+01 | 6.09E-05 | 2.82E-04 |
| D-Mannose | 1.88E+00 | 1.39E+01 | 6.90E-05 | 3.11E-04 |
| 3-Hydroxydodecanoic acid | -1.03E+00 | 1.62E+01 | 7.52E-05 | 3.29E-04 |
| Theophylline | -3.41E+00 | 1.43E+01 | 8.94E-05 | 3.80E-04 |
| L-Serine | -8.09E-01 | 1.46E+01 | 1.25E-04 | 5.18E-04 |
| Uridine | -8.82E-01 | 1.25E+01 | 1.95E-04 | 7.86E-04 |
| L-Asparagine | -7.43E-01 | 1.48E+01 | 2.21E-04 | 8.69E-04 |
| Maltotriose | 7.26E-01 | 8.27E+00 | 1.55E-03 | 5.93E-03 |
| D-Aspartic acid | -7.32E-01 | 1.47E+01 | 2.12E-03 | 7.57E-03 |
| Phenol | 9.09E-01 | 1.41E+01 | 2.13E-03 | 7.57E-03 |
| Dihydrouracil | -9.94E-01 | 1.26E+01 | 2.19E-03 | 7.63E-03 |
| Cholic acid | 1.92E+00 | 1.31E+01 | 4.50E-03 | 1.53E-02 |
| Saccharin | -1.31E+00 | 1.28E+01 | 6.29E-03 | 2.09E-02 |
| L-Cystine | 9.58E-01 | 1.30E+01 | 7.74E-03 | 2.52E-02 |
| L-Kynurenine | 1.15E+00 | 1.22E+01 | 9.27E-03 | 2.89E-02 |
| Sphingosine | -1.10E+00 | 1.55E+01 | 1.02E-02 | 3.11E-02 |
| Erucic acid | -1.29E+00 | 1.45E+01 | 1.14E-02 | 3.42E-02 |
| Dopamine | 1.65E+00 | 1.28E+01 | 1.20E-02 | 3.52E-02 |
| 2,4-Dinitrophenol | 7.37E-01 | 1.53E+01 | 1.44E-02 | 4.02E-02 |
| Iminodiacetic acid | 6.34E-01 | 1.25E+01 | 1.59E-02 | 4.34E-02 |
| Mevalonic acid | -8.45E-01 | 1.17E+01 | 3.64E-02 | 9.60E-02 |
| D-Threitol | 1.22E+00 | 1.42E+01 | 4.23E-02 | 1.05E-01 |
| Riboflavin | -7.59E-01 | 1.12E+01 | 4.25E-02 | 1.05E-01 |
| Sunitinib | -6.77E-01 | 2.04E+01 | 4.87E-02 | 1.16E-01 |
